# Marum Verum

**Published:** 1887-10

**Authors:** 


MARUM VERUM: Little children, becoming emaciated, are helped by *Marum*, if they have a jerking hiccough after nursing, and belching without bringing anything up. The same is often beneficial in the crying or the diarrhœa of little children.—*Pehrson.*

